# Ultrasound-assisted gatifloxacin delivery in mouse cornea, *in vivo*

**DOI:** 10.1038/s41598-019-52069-w

**Published:** 2019-10-29

**Authors:** Uk Jegal, Jun Ho Lee, Jungbin Lee, Hyerin Jeong, Myoung Joon Kim, Ki Hean Kim

**Affiliations:** 1 Department of Mechanical Engineering, Pohang University of Science and Technology, 77 Cheongam-ro, Nam-gu, Pohang, Gyeoungbuk, 37673 Republic of Korea; 2 Division of Integrative Biosciences and Biotechnology, Pohang University of Science and Technology, 77 Cheongam-ro, Nam-gu, Pohang, Gyeoungbuk, 37673 Republic of Korea; 3 Department of Ophthalmology, Ulsan College of Medicine, Asan Medical Center, 88 Olympic-ro 43-gil, Songpa-gu, Seoul, 05505 Republic of Korea

**Keywords:** Biological fluorescence, Cellular imaging, Corneal diseases, Nonlinear optics, Multiphoton microscopy

## Abstract

Gatifloxacin is a 4th generation fluoroquinolone antibiotic used in the clinic to treat ocular infection. One limitation of gatifloxacin is its relatively poor corneal penetration, and the increase of its trans-corneal delivery would be beneficial to reduce the amount or frequency of daily dose. In this study, ultrasound treatment was applied to enhance the trans-corneal delivery of gatifloxacin without damage. Experiments were conducted on mouse eyes in *ex vivo* and *in vivo* conditions. Ultrasound waves with 1 MHz in frequency, 1.3 W/cm^2^ in intensity were applied onto the mouse cornea for 5 minutes, and then gatifloxacin ophthalmic solution was instilled and left there for 10 minutes. 3D gatifloxacin distribution in the cornea was measured by two-photon microscopy (TPM) imaging based on its intrinsic fluorescence. Longitudinal TPM imaging of ultrasound treated mouse corneas showed the increase of initial gatifloxacin intensities on the corneal surface compared to untreated mouse corneas by 67%, and then the increased gatifloxacin delivery into the cornea from the surface at later time. The delivered gatifloxacin in the corneal epithelium stayed longer in the ultrasound treated corneas than in the untreated corneas. The enhanced trans-corneal delivery and extended stay of gatifloxacin in the mouse cornea by ultrasound treatment could be beneficial for therapeutic effects. This study demonstrated the detail process of enhanced trans-corneal gatifloxacin delivery by ultrasound treatment.

## Introduction

Gatifloxacin is one of fourth-generation fluoroquinolone antibiotics with outstanding antibacterial effects and wide antibacterial spectrum^1–4^. Retention of moderate concentration of antibiotics including gatifloxacin within the cornea is important to get desired therapeutic effects in keratitis treatment and postoperative infection prevention^5,6^. However, gatifloxacin showed relatively poor penetration into the cornea compared to other fluoroquinolone antibiotics such as moxifloxacin, levofloxacin^3,5,7^. Eye’s physiological and anatomical features make gatifloxacin delivery into the cornea difficult^3,8,9^. Topically administered gatifloxacin will be easily absorbed into the conjunctival sacs and this has a negative effect on corneal absorption^6^. The tear film clearance system in the pre-ocular region also reduces corneal absorption. The cornea, consisting of three layers, has poor permeability characteristics. The corneal epithelium is lipophilic, which means the barrier for hydrophilic compounds^10^. The stroma is hydrophilic, which means the barrier for lipophilic ones. Therefore, the cornea is considered as an efficient barrier to the absorption of both hydrophilic and lipophilic compounds. Corneal penetration of gatifloxacin, formulated as eye drops, is estimated to be approximately 5%^2^. Due to the low corneal permeability of gatifloxacin, large dose or frequent application is needed in clinic, possibly resulting in side effects such as dyspnoea, arrhythmia and pathoglycemia^2,9,11,12^. Therefore, the increase of corneal penetration of gatifloxacin would be desirable to achieve therapeutic effects with minimal daily dose and frequency^2,13^. Methods to enhance gatifloxacin delivery into the cornea would be important to get desired antibacterial effects. Ultrasound waves have been used to increase the corneal penetration of drugs such as dexamethasone sodium phosphate and fluorescein without tissue damage^8,14^. Ultrasound waves induce cavitation microbubbles in the coupling liquid, and the oscillation and collapse of microbubbles make pores into the cell membrane and enhance the delivery of large molecules^15–21^. The porosity and permeability of cell membrane can be transiently increased. Previously, several studies were conducted to demonstrate the enhancement of corneal drug delivery via ultrasound treatment^8,14,22^. A previous *in vivo* study showed that 880 kHz ultrasound with the intensity of 0.56 W/cm^2^ and 5 min exposure resulted in 10.6 times increase of corneal permeability for a drug mimicking sodium fluorescein^14^. Another *in vivo* study showed that ultrasound treatment at 400 kHz increased the permeation of riboflavin through the corneal epithelium and into the corneal stroma, compared to untreated controls^8^. The distribution of drugs within the cornea was measured by using confocal microscopy (CM) based on intrinsic fluorescence of drugs and drug models^22^. In case of gatifloxacin, two-photon microscopy (TPM) was used previously for the visualization of distribution in the cornea based on its intrinsic fluorescence^3^. TPM used a near infrared excitation wavelength instead of ultra-violet (UV) wavelength, where the single-photon excitation peak of gatifloxacin is located, by utilizing nonlinear two-photon excitation process^3^.

In this study, ultrasound-assisted delivery of gatifloxacin into the mouse cornea was tested in both *ex vivo* and *in vivo* conditions. First, the ultrasound intensity applicable to the cornea without damage was determined experimentally. Then, the distribution of topically administered gatifloxacin in the mouse cornea was measured by 3D TPM imaging based on intrinsic fluorescence of gatifloxacin. The enhancement of gatifloxacin delivery into the mouse cornea via ultrasound was measured by comparing gatifloxacin distribution in between the ultrasound treated and untreated corneas. Temporal changes of gatifloxacin distribution in the mouse cornea were measured by time-lapse TPM imaging and analysis.

## Results

### Optimization of ultrasound intensity for the mouse cornea treatment

**Figure 1:**
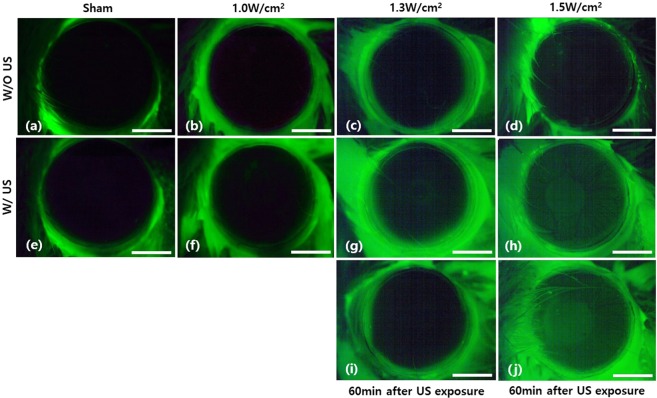
En-face images of *in vivo* mouse corneas stained with 0.25% fluorescein solution before and after ultrasound treatment of various intensities. (**a**–**d**) and (**e**–**h**) En-face images of fluorescein stained mouse corneas before and just after the ultrasound treatment, respectively. Ultrasound intensities for (**e**–**h**) were 0 W/cm^2^ (Sham), 1.0 W/cm^2^, 1.3 W/cm^2^, 1.5 W/cm^2^, respectively. (**i**,**j**) En-face mouse cornea images In 60 min after the ultrasound treatment. Ultrasound intensities for (**i**) and (**j**) were 1.3 W/cm^2^ and 1.5 W/cm^2^, respectively. The scale bar indicates 1 mm.

Ultrasound intensity, which could be applied without damage, was measured by treating mouse corneas with various ultrasound intensities and by examining them with fluorescein staining. Various ultrasound intensities of 0 W/cm^2^, 1.0 W/cm^2^, 1.3 W/cm^2^, and 1.5 W/cm^2^ were applied for 5 min. Fluorescein staining and fluorescence microscopy examination was conducted on the mouse corneas both before and after the ultrasound treatment. The examination results are shown in Fig. 1. In case of sham (0 W/cm^2^), fluorescence was not detected in the cornea both before and after the ultrasound treatment owing to no damage induced. Fluorescence was seen only from the corneal surrounding, where some fluorescein remained even after washing. With the increase of ultrasound intensities, some fluorescence was detected in the cornea. In case of 1.0 W/cm^2^ ultrasound intensity, not much fluorescence was detected in the cornea after the ultrasound treatment. However, in case of 1.3 W/cm^2^ and 1.5 W/cm^2^ ultrasound intensities, some uniform fluorescence appeared in the corneas after the ultrasound treatment and its level increased with ultrasound intensities. The mouse cornea, treated with 1.5 W/cm^2^ ultrasound intensity, showed a localized fluorescein stain even before the ultrasound treatment. However, this localized staining did not affect the experiment result much, because the cornea showed uniform fluorescein distribution after the ultrasound treatment. Although the mouse corneas treated with both 1.3 W/cm^2^ and 1.5 W/cm^2^ ultrasound intensities showed some damages, these ultrasound-induced damages could be temporary and would be healed after some time. To test whether these damages were temporary, the same corneas were tested with fluorescein stain again in 1 hour later and the results are shown in Fig. 1(I,j). In case of 1.3 W/cm^2^ intensity, the mouse cornea did not show fluorescence in the second fluorescein stain indicating recovery. However, the mouse cornea treated with 1.5 W/cm^2^ showed fluorescence in the second test indicating the damage was not temporary. From the results, the maximum ultrasound intensity applicable to the mouse cornea without damage was measured to be 1.3 W/cm^2^. In addition to fluorescein staining and fluorescence microscopy examination of the mouse cornea, temperature increase by ultrasound treatment was measured during the ultrasound application to assess potential thermal damage. In case of 1.3 W/cm^2^ ultrasound intensity, temperature increased up to 0.5 °C maximum. The level of temperature increase was below the maximum allowed temperature increase of 1.5 °C by thermal safety requirements^23^. Therefore, potential thermal damage with 1.3 W/cm^2^ ultrasound intensity was negligible under the current ultrasound treatment protocol.

### Ultrasound assisted delivery of gatifloxacin into the mouse cornea, *ex vivo*

**Figure 2:**
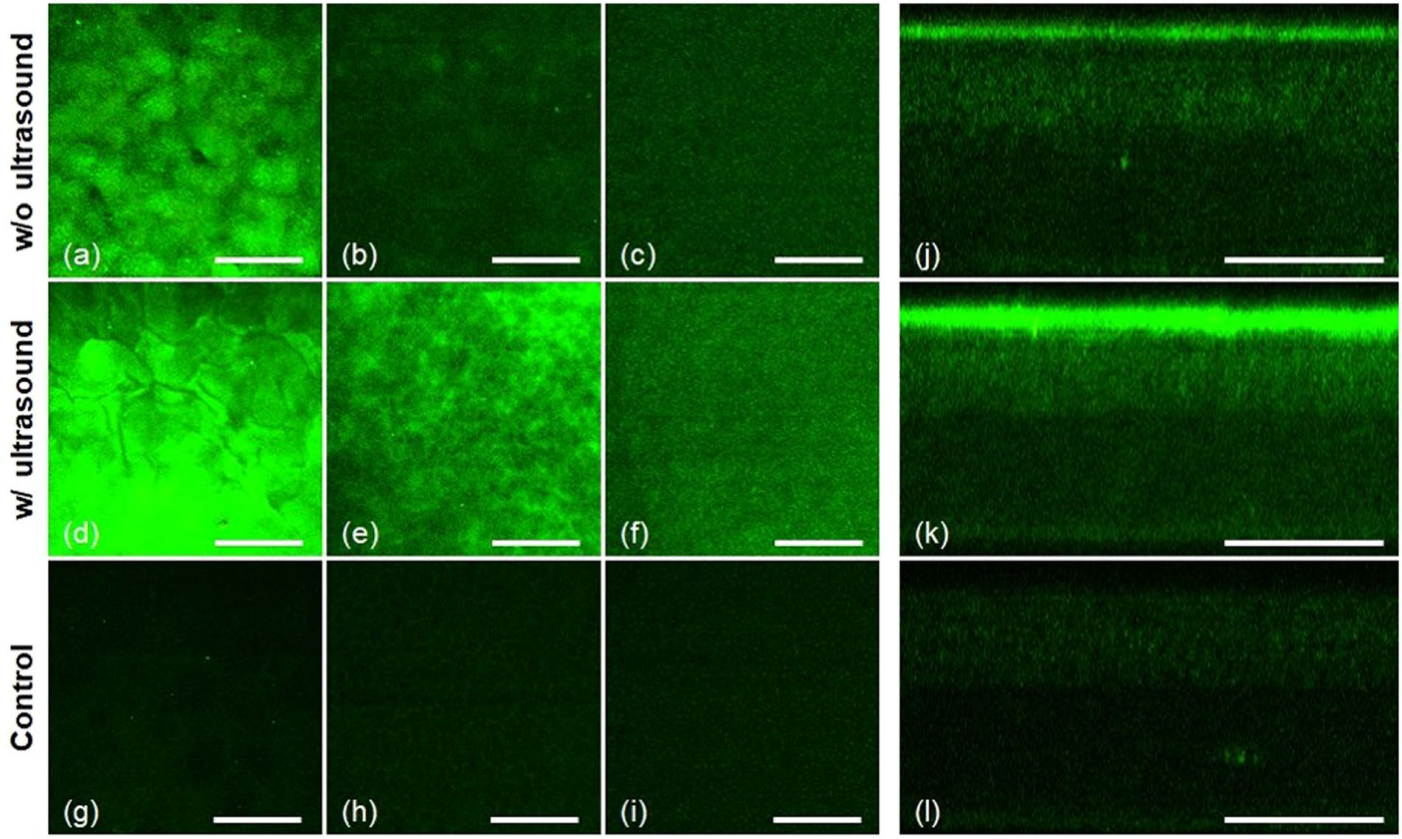
3D TPM images of gatifloxacin instilled *ex vivo* mouse corneas without (**a**–**c** and **j**) and with (**d**–**f** and **k**) ultrasound treatment. TPM images were acquired after 10-minute gatifloxacin incubation. (**a**–**c**) and (**d**–**f**) En-face TPM images of the mouse corneas at 3 different depths without and with ultrasound treatment, respectively (Supplementary Video 1, 2). En-face TPM images on the surface, just below the surface (12 µm deep from the surface), and in the basal epithelium (46 µm deep from the surface) are presented. (**g**–**i**) En-face auto-fluorescence (AF) TPM images of the mouse cornea at 3 different depths (Supplementary Video 3). (**j**) and (**k**) Cross-sectional TPM images of the gatifloxacin instilled mouse corneas without and with ultrasound, respectively. (**l**) A cross-sectional AF TPM images of the mouse cornea. The scale bar indicates 100 µm.

Ultrasound assisted gatifloxacin delivery into the mouse cornea was tested in the *ex vivo* condition first. 3D TPM images of gatifloxacin instilled mouse corneas without and with ultrasound treatment after 10-minute incubation are shown in Fig. 2 (Supplementary Video 1, 2). Three en-face TPM images showing the corneal epithelium layers on the surface, just below the surface (12 µm deep), and in the basal epithelium (46 µm deep), and a cross-sectional TPM image are presented in each condition. TPM images of gatifloxacin instilled mouse corneas showed gatifloxacin distribution with respect to corneal epithelial cells. Epithelial cells were weakly visible partly owing to non-uniform distribution of gatifloxacin and their intrinsic auto-fluorescence (AF) expression. TPM images of an *ex vivo* mouse cornea without gatifloxacin are presented as well in order to show the level of AF. Without ultrasound treatment, TPM images of the gatifloxacin instilled mouse cornea showed relatively strong gatifloxacin fluorescence on the corneal surface only. The en-face image on the corneal surface showed high gatifloxacin fluorescence, and the image a little bit below the corneal surface showed much weaker fluorescence around corneal epithelial cells. Corneal epithelial cells in this layer were visible based on both gatifloxacin fluorescence and AF, because TPM image based on AF showed similar cellular structures with the lower intensities than the current one. In addition to cellular structure, there were broadly distributed fluorescence signals visible in the image and these might be from gatifloxacin, penetrated through the corneal surface. The cross-sectional TPM image of the gatifloxacin instilled mouse cornea without ultrasound treatment showed the depth distribution of gatifloxacin fluorescence. There was strong fluorescence on the corneal surface, weak fluorescence in the corneal epithelium below the surface, and almost no fluorescence in the stroma. Fluorescence levels in the corneal epithelium below the surface were a little bit higher than that of the mouse cornea without gatifloxacin. This result indicated that most of gatifloxacin stayed on the corneal surface and a little of gatifloxacin penetrated through the corneal surface after 10-minute incubation without ultrasound treatment and this result was consistent with a previous report^3^. With ultrasound treatment, TPM images of the gatifloxacin instilled mouse cornea showed the increased fluorescence both on and just below the corneal surface compared to those without ultrasound treatment. The en-face TPM image of the corneal surface showed very strong gatifloxacin fluorescence so that the image was saturated in the current min/max scale for display. The en-face TPM images both at 12 µm below the surface and in the basal epithelium showed quite strong fluorescence around corneal epithelial cells, indicating penetration of gatifloxacin across the corneal surface. The cross-sectional TPM image of the ultrasound treated mouse cornea showed much stronger fluorescence both on the corneal surface and inside the epithelium compared to those of the untreated mouse cornea. This indicated the increased penetration of gatifloxacin into the cornea through the barrier on the corneal surface via ultrasound treatment.

### Ultrasound-assisted gatifloxacin delivery in the mouse cornea, *in vivo*

**Figure 3:**
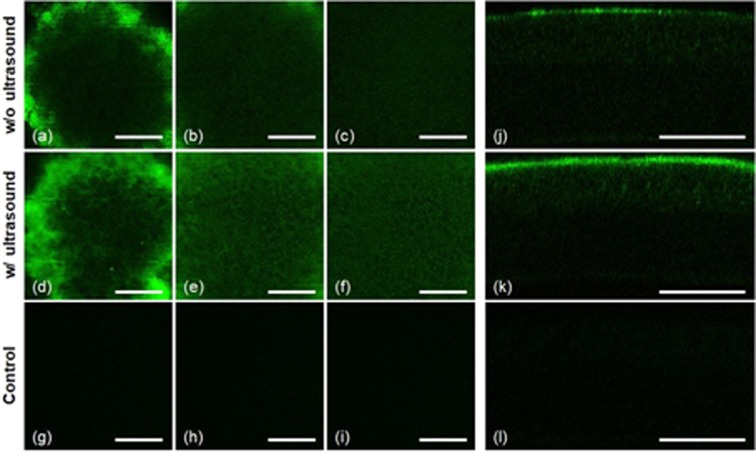
TPM images of gatifloxacin instilled *in vivo* mouse corneas without (**a**–**c** and **j**) and with (**d**–**f** and **k**) ultrasound treatment. TPM images were acquired after 10-minute gatifloxacin incubation. (**a**–**c**) and (**d**–**f**) En-face TPM images of the mouse corneas at 3 different depths without and with ultrasound treatment, respectively (Supplementary Video 3, 4). En-face TPM images at 3 different layers of the superficial epithelium (first column), middle epithelium (second column), and basal epithelium (third column) are presented. (**g**–**i**) En-face auto-fluorescence (AF) TPM images of the mouse cornea. (**j**,**k**) Cross-sectional TPM images of the gatifloxacin instilled mouse corneas without and with ultrasound, respectively. (**l**) A cross-sectional AF TPM images of the mouse cornea. The scale bar indicates 100 µm.

After the confirmation of ultrasound-assisted enhanced gatifloxacin delivery in the *ex vivo* mouse corneas, ultrasound-assisted trans-corneal delivery of gatifloxacin was measured in the *in vivo* condition. All the experimental procedures were conducted in live mice under gas anesthesia. 3D TPM images of gatifloxacin distribution in the mouse cornea with ultrasound treatment were compared with the ones without ultrasound treatment, and results are shown in Fig. 3. Gatifloxacin distributions after 10-minute incubation were compared. Without ultrasound treatment (Supplementary Video 3), TPM images of the gatifloxacin instilled mouse cornea showed strong gatifloxacin fluorescence on the corneal surface and not much fluorescence below the surface. The en-face TPM image on the first superficial layer showed a band of strong and relatively uniform fluorescence around the center. This fluorescent band was generated from gatifloxacin on the corneal surface which was curved with respect to the imaging plane in the live mouse. The strong fluorescent band indicated high gatifloxacin concentration on the corneal surface. Epithelial cells below the corneal surface were shown inside the strong fluorescent band of the first layer image. The epithelial cells were not clearly resolved owing to additional hazy fluorescence distributed around the cells, and this indicated penetration of some gatifloxacin across the corneal surface. With ultrasound treatment (Supplementary Video 4), the en-face TPM image of the first superficial layer showed the increased fluorescence both on the surface band and in the epithelial cell layer below the surface compared to the ones without ultrasound treatment. The increased fluorescence on the corneal surface was clear in the cross-sectional TPM image showing the thicker band of gatifloxacin fluorescence compared to the one of without ultrasound treatment. The en-face TPM image of the second superficial layer showed increased fluorescence around the epithelial cells below the surface as well.

### Quantitative analysis of gatifloxacin distribution in the mouse cornea, *in vivo*

**Figure 4:**
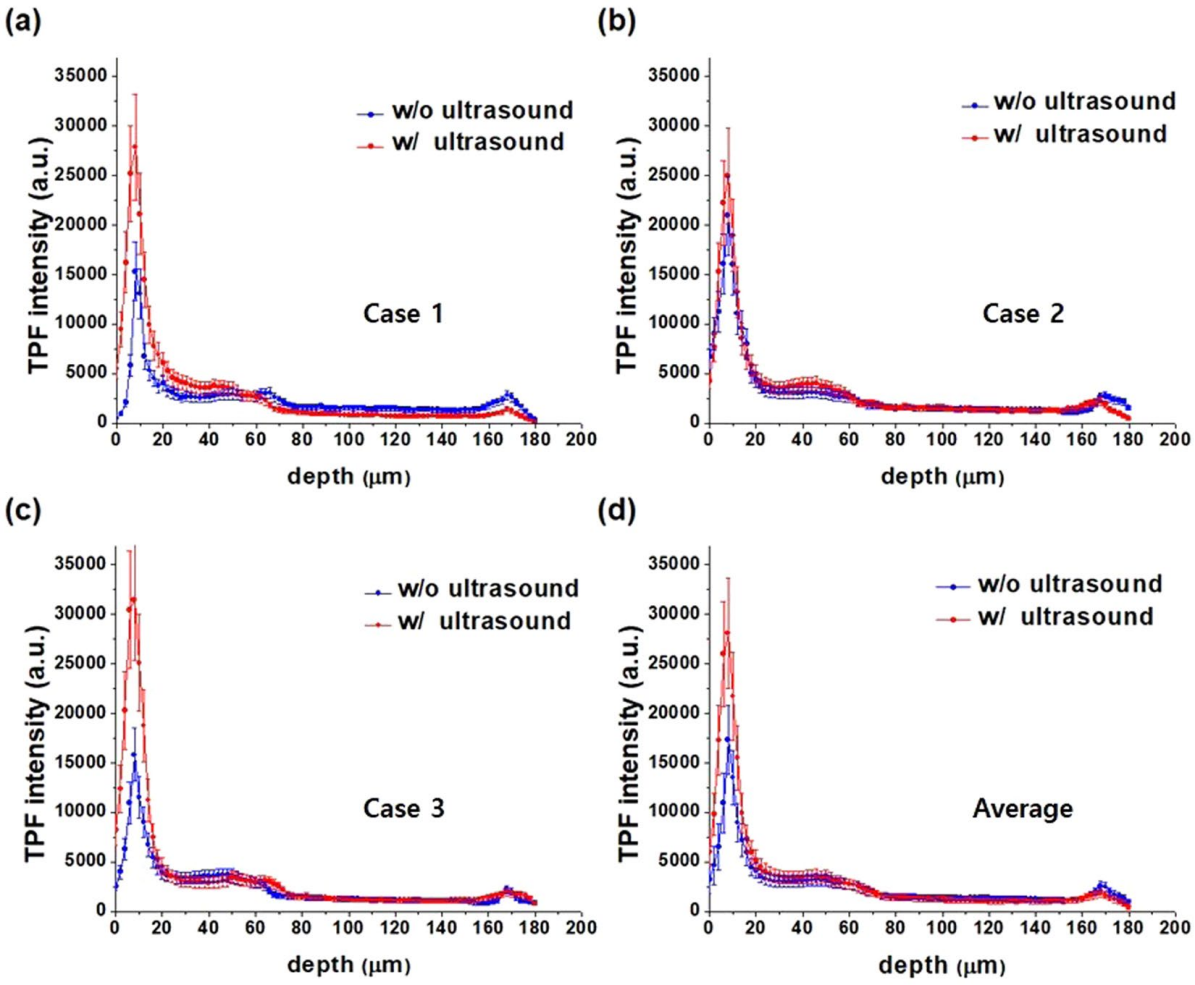
Depth profiles of gatifloxacin intensity in the mouse cornea, *in vivo*. (**a**–**c**) Depth profiles of gatifloxacin intensity in 3 different mouse corneas without and with ultrasound treatment after 10-minute gatifloxacin incubation. (**d**) Averaged depth profiles of gatifloxacin intensity in the mouse cornea without and with ultrasound treatment.

TPM images of gatifloxacin distribution in the ultrasound treated mouse cornea after 10-minute incubation showed the significant increase of gatifloxacin fluorescence on the corneal surface compared to those without ultrasound treatment. The increased gatifloxacin fluorescence was analyzed quantitatively by calculating the depth profile of gatifloxacin fluorescence intensity. Intensity depth profiles in the gatifloxacin instilled mouse cornea had the contributions of both gatifloxacin fluorescence and AF. So, the depth profiles of gatifloxacin fluorescence only were obtained by subtracting the AF depth profile. *In vivo* experiments were conducted three times, and depth profiles of gatifloxacin intensity in the individual mouse corneas and an averaged depth profile of all the three cases are presented in Fig. 4. Depth profiles of gatifloxacin intensity after 10-minute incubation showed peak intensities on the corneal surface in the both cases of without and with ultrasound treatment. These intensity peaks indicated high gatifloxacin density on the corneal surface after 10-minute incubation, although gatifloxacin density on the corneal surface would decrease later via diffusion. With ultrasound treatment, gatifloxacin intensities on the corneal surface were higher than those without treatment in all the three cases. Ultrasound treatment increased gatifloxacin density on the corneal surface either by increasing the permeability of corneal cells or by increasing the intercellular space. Beside the corneal surface, gatifloxacin intensities in the corneal epithelium increased slightly with ultrasound treatment. The difference of gatifloxacin intensities in the corneal epithelium between with and without ultrasound treatment was not much, although it was clearly shown in the en-face images. This might be because majority of image pixels in the epithelium showed intra-cellular structures such as the cell nucleus and these pixels did not have much intensities. The increase of peak gatifloxacin intensity on the corneal surface by ultrasound treatment was approximately 67% on average.

### Temporal changes of gatifloxacin distribution in the *in vivo* mouse cornea after ultrasound treatment

**Figure 5:**
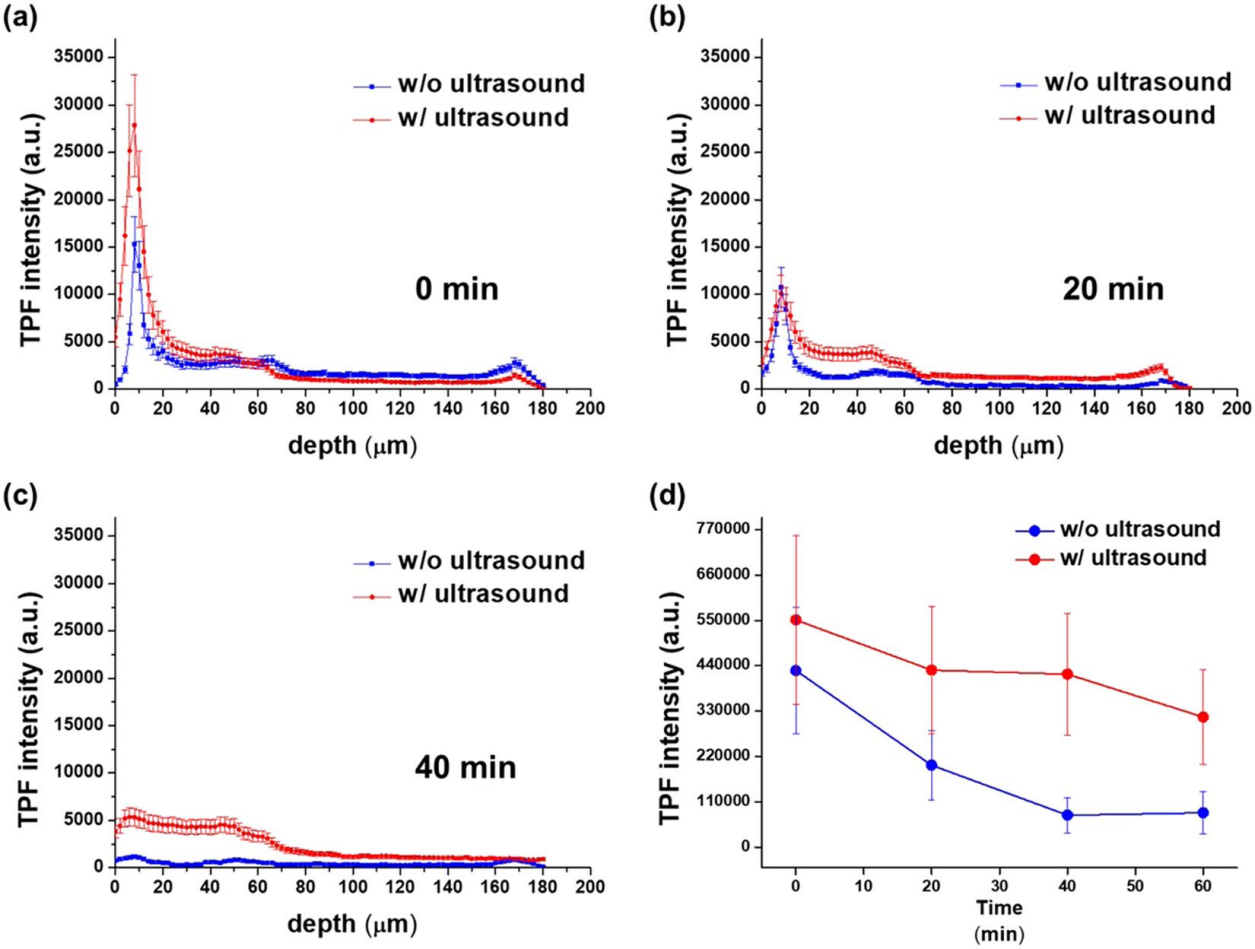
Temporal changes of gatifloxacin depth profiles in the *in vivo* mouse cornea without and with ultrasound treatment. (**a**–**c**) Depth profiles of averaged gatifloxacin intensity in the ultrasound treated and untreated mouse corneas in 0 minute, 20 minutes, and 40 minutes after 10-minute gatifloxacin incubation. (**d**) Temporal changes of total gatifloxacin fluorescence intensity in the ultrasound treated and untreated mouse corneas after the gatifloxacin incubation.

3D TPM imaging conducted right after 10-minute incubation showed that ultrasound treatment mainly increased gatifloxacin density on the corneal surface. This increased gatifloxacin on the corneal surface might help diffusion of more gatifloxacin into the cornea later. Temporal changes of gatifloxacin distribution in the mouse cornea were measured by conducting longitudinal TPM imaging at every 20 minutes for 60 minutes total starting from right after incubation: 0, 20, 40, and 60 minutes post incubation. Depth profiles of average gatifloxacin fluorescence intensity in a mouse cornea at different time points are shown in Fig. 5. The depth profiles in 0, 20, and 40 minutes post incubation are shown in Fig. 5(a–c), and the temporal change of total gatifloxacin intensity in the cornea is shown in Fig. 5(d). At each time point, depth intensity profiles in both the ultrasound treated and untreated corneas are shown. Right after the incubation, the gatifloxacin intensity profiles showed that peak intensities were on the corneal surface in both the ultrasound treated and untreated corneas and the one in the treated cornea had the higher peak intensity than the one in the untreated cornea. Other than the corneal surface, gatifloxacin intensities in the corneal epithelium were slightly higher in the ultrasound treated cornea than in the untreated cornea. In 20 minutes post incubation, gatifloxacin intensity profile in the ultrasound treated cornea showed different intensity changes on the corneal surface and in the corneal epithelium. Gatifloxacin intensities in the corneal epithelium of the ultrasound treated cornea remained the same or increased, while the ones on the corneal surface decreased significantly. On the other hand, gatifloxacin intensities in the untreated cornea decreased both on the surface and in the epithelium in 20 minutes post incubation compared to the ones in 0 minute post incubation owing to diffusion of gatifloxacin. In 40 minutes post incubation, gatifloxacin intensities inside the corneal epithelium remained or decreased slowly in the ultrasound treated cornea. On the other hand, the intensities in the untreated cornea were very low in the corneal epithelium. These depth profiles of gatifloxacin intensity at different time points indicated that ultrasound treatment increased gatifloxacin density on the corneal surface significantly in 0 minute post incubation and maintained gatifloxacin in the corneal epithelium longer than the case without treatment. Temporal changes of total gatifloxacin intensity in the *in vivo* mouse cornea showed this trend. Total gatifloxacin intensity in the mouse cornea was calculated by integrating 3D TPM images. Total gatifloxacin fluorescence intensity in the mouse cornea had maximum values right after the incubation and decreased monotonically with time in both the ultrasound treated and untreated mouse corneas. However, the decay rate of total gatifloxacin fluorescence intensity was different. Total gatifloxacin intensity decreased slower in the ultrasound treated cornea than in the untreated cornea. The ratio of total gatifloxacin intensity in the ultrasound treated cornea with respect to the untreated cornea was 127%, 148%, 179%, 164% at the time points of 0 minute, 20 minutes, 40 minutes, 60 minutes post incubation, respectively. P-value for the total gatifloxacin intensity ratio was 0.007, 0.013, 0.0002, 0.0001 at the time points of 0 minute, 20 minutes, 40 minutes, 60 minutes post incubation, respectively (P < 0.05). Total gatifloxacin intensity in the ultrasound treated cornea was higher than the one in the untreated cornea by 27% on average right after the incubation. The ratio of total gatifloxacin intensity increased with time. Approximately half of the initial total gatifloxacin intensity was still maintained in 60 minutes later in the ultrasound treated cornea. Ultrasound treatment appeared not only to increase the gatifloxacin delivery into the cornea but also to maintain gatifloxacin concentration inside the cornea for the extended time duration.

## Discussions and Conclusions

Ultrasound treatment was used to increase the trans-corneal delivery of gatifloxacin ophthalmic solution. Ultrasound waves with 1 MHz in frequency were applied onto the mouse cornea for 5 minutes, and then gatifloxacin ophthalmic solution was topically instilled right after the ultrasound treatment. After 10-minute incubation, gatifloxacin distribution in the mouse cornea was imaged by TPM. Temporal changes of gatifloxacin distribution in the mouse cornea were visualized by longitudinal TPM imaging every 20 minutes for 60 minutes. Effects of ultrasound treatment on gatifloxacin trans-corneal delivery were assessed by comparing the gatifloxacin distributions in the ultrasound treated and untreated mouse corneas. In the untreated mouse cornea, TPM showed an intensity peak on the corneal surface and relatively low intensities in the corneal epithelium right after the gatifloxacin incubation. The initial gatifloxacin distribution in the cornea indicated that the major barrier of gatifloxacin delivery was the superficial corneal epithelium. Later, gatifloxacin intensity on the corneal surface decreased quickly and the ones in the corneal epithelium decreased also as gatifloxacin in the cornea diffused. Gatifloxacin intensities in the stroma were always lower than those in the epithelium, probably owing to relatively fast gatifloxacin diffusion in the hydrophilic stroma. In the ultrasound treated mouse cornea, TPM showed several changes in gatifloxacin distribution. Initial gatifloxacin peak intensity on the corneal surface was 67% higher in the ultrasound treated cornea than in the untreated cornea. The increase of peak intensity could be owing to break-up of the hydrophobic barrier and the more accumulation of hydrophilic gatifloxacin. Initial gatifloxacin intensities in the corneal epithelium were slightly higher in the ultrasound treated cornea than in the untreated cornea. Then, the intensities in the corneal epithelium decreased much slowly in the ultrasound treated cornea compared to the ones in the untreated cornea. The mechanism is not clear but gatifloxacin might be trapped in either intra- or inter-cellular spaces owing to the increased permeability of cells caused by ultrasound treatment. These effects of ultrasound treatment could be positive to therapeutic effects of gatifloxacin, because topically instilled gatifloxacin remained in the cornea for the extended time. Further study would be needed to measure the therapeutic effects of ultrasound waves by using infection animal models. These effects of ultrasound treatment might be universal for other drugs with similar pharmacokinetic properties as gatifloxacin. Ultrasound treatment is known to be a safe method by temporally increasing the permeability of cell membrane. Therefore, the ultrasound assisted gatifloxacin delivery method could be useful for clinical applications.

The mechanisms for ultrasound mediated drug delivery have been studied previously^8,14,23–29^. Ultrasound waves generate cavitation bubbles and streaming within the coupling medium by inducing a pressure gradient. There are two types of cavitation: stable and inertial. Stable cavitation is the uniform pulsation of bubbles over many pressure cycles without collapse, and the oscillation of these bubbles produce mechanical stresses on the nearby cells^8,24–26^. The bubble oscillation also generates a microscale circulatory flow around the bubble, called as microstreaming. Microstreaming may cause the membrane rupture of nearby cells by inducing shear stresses^14,23,27^. In the inertial cavitation, microbubbles grow within a few pressure cycles and collapse. Then, shock waves and microjets are generated during the bubble collapse and can pore the cell membrane^8,24,27,28^. Cavitation and streaming effects are considered as the main mechanism of ultrasound wave mediated enhanced drug delivery in general^14,23^. Ultrasound parameters used in this study were in the range of cavitation generation^23,24^. The enhanced gatifloxacin intensity on the corneal surface in the ultrasound treated mouse model could be explained by the cavitation and streaming effects. Ultrasound waves generate a bluk flow in the coupling medium called acoustic streaming. Acoustic streaming is able to move ions and small molecules, and shearing and streaming forces may dirupt cell walls. Acoustic streaming may contribute to the enhancement of drug delivery^27,29^. Besides the cavitation and streaming effects, ultrasound waves induce temperature increase due to absorption of ultrasound energy by tissues. Although temperature increase was correlated with the enhancement of drug permeability, too much increase can damage ocular tissues^23^. The effects of ultrasound waves on temperature were negligible in this study, because the temperature increase was measured to be less than 0.5 °C maximum.

TPM imaging was a direct method for measuring gatifloxacin distribution in the cornea based on gatifloxacin intrinsic fluorescence. TPM visualized both gatifloxacin distribution and cellular structure of the mouse cornea simultaneously and showed the temporal changes of gatifloxacin distribution in the cornea. Simultaneous visualization of the cellular structure and gatifloxacin distribution in the mouse cornea allowed to analyze the local distribution of gatifloxacin with respect to corneal epithelial cells, and the further study could elucidate the pathways of gatifloxacin delivery into the corneal surface and inter- or intra-distribution of gatifloxacin. Longitudinal TPM imaging of gatifloxacin distribution visualized additional effects of ultrasound treatment on the gatifloxacin distribution in the cornea. Microscopic imaging methods based on the intrinsic fluorescence of drugs might be useful to study corneal drug delivery pathway.

In the current study, gatifloxacin was instilled onto the mouse cornea right after the ultrasound treatment. The enhancement of gatifloxacin delivery by this method would be less than the case of prior gatifloxacin instillation and ultrasound treatment using the gatifloxacin solution as ultrasound coupling medium. However, previous ultrasound study showed that the increased permeability by ultrasound treatment lasted longer than 10 minutes^20^. Therefore, the current treatment protocol would be still effective. Ultrasound treatment with gatifloxacin would need additional device development to hold gatifloxacin solution during ultrasound treatment, and this method may not be not practical for human due to increased complication of the procedure.

The efficiency of trans-corneal gatifloxacin delivery by ultrasound treatment was lower compared to other previous studies on the ultrasound assisted delivery of other compounds. Our study showed approximately 27–80% enhanced gatifloxacin delivery into the cornea with ultrasound treatment, while others showed the enhancement from 43% to 126%^8,14,24^. This difference could be explained mainly by using different measurement methods. In this study, 3D TPM imaging was conducted to measure the amount of gatifloxacin in the cornea and total gatifloxacin fluorescence intensity was obtained by integrating the 3D TPM image. However, this method tends to under-estimate the amount of gatifloxacin delivery because fluorescence intensity is affected not only by drug concentration but also by other parameters such as light scattering and absorption. Fluorescence intensity usually goes down as the imaging plane goes deep into the cornea even with the uniform concentration. TPM is less sensitive in the measurement of gatifloxacin than other measurement methods such as high-performance liquid chromatography (HPLC) due to additional AF noise so that gatifloxacin measurement in the deep corneal layers such as the stroma may not be accurate. Effects of ultrasound treatment on the gatifloxacin trans-corneal delivery were measured by TPM imaging of the mouse cornea in less than 1 hour post incubation. Other previous *in vivo* studies measured the concentration of drugs in aqueous humor in over 1 hour^8,20^, Thus, different measurement time may affect the enhancement measurement of gatifloxacin trans-corneal delivery.

Damage of the cornea by ultrasound treatment was assessed by fluorescein staining and fluorescence microscopy examination. The examination with fluorescein was to check the integrity of the superficial epithelium and its barrier function. With the current ultrasound intensity, the barrier function was restored within 1 hour post ultrasound treatment. Temperature increase by ultrasound treatment was measured. The level of temperature increase was only 0.5 °C maximum in the current treatment protocol, and this level was below the limit of thermal safety requirement which is 1.5 °C^23^. In addition to the superficial corneal epithelium, other parts of the cornea such as the endothelium and limbus could be damaged by ultrasound treatment. Damage of the endothelium was assessed partially by follow-up visual examination of the corneal clarity for a few days post experiment. Good corneal clarity was maintained in all the mice during the follow-up examination, indicating the intactness of the endothelium function. Stem cells in the limbus could be vulnerable to ultrasound treatment. The intactness of the corneal function was assessed partially by the follow-up visual examination in the current study, but specific parameters associated with limbal stem cell health were not examined. However, damage of limbal stem cells can be avoided by applying ultrasound waves locally onto the cornea only.

Balb/c mouse models were used to demonstrate the ultrasound assisted enhanced trans-corneal gatifloxacin delivery. Although mouse corneas do the same function as human corneas by refracting incoming light for vision, mouse corneas are slightly different from human ones in terms of thickness and composition. Mouse corneas are thinner than human ones: thickness of the mouse and human cornea is approximately 170 μm and 500 μm^30^, respectively. Mouse corneas have been known to have different layer composition from human ones. Human corneas are composed of three different layers and two interfaces: the corneal epithelium, anterior limiting lamina (Bowman’s layer), stroma, posterior limiting lamina (Descemet’s membrane), and endothelium^31,32^. Mouse corneas have been known not to have the anterior limiting lamina (Bowman’s layer). However, some previous studies by Zhang *et al*. and Reichard *et al*. showed the existence of the anterior limiting lamina (Bowman’s layer) in the C57BL/6 and BALB/c mice^33,34^. Thus, mouse corneas also have five layers like human corneas. The anterior limiting lamina (Bowman’s layer) in the mouse cornea was not easily detectable because it is thinner than the one in the human cornea and it shrinks with fixation. The Bowman’s layer was not a critical factor in the trans-corneal delivery of gatifloxacin, and the major barrier was the superficial corneal epithelium. There are two more differences between mouse and human corneas. In mouse corneas, the center part is thinner than the periphery while human corneas are opposite to those of mouse. The thickness proportion of the corneal epithelium in the mouse cornea is higher than that in the human cornea^35^. With the listed differences in between mouse and human corneas, the degree of enhancement of gatifloxacin trans-corneal delivery might be different. However, if trans-corneal drug delivery enhancement by ultrasound was shown in the mouse cornea, we can expect trans-corneal drug delivery enhancement in the human cornea whose layers are arranged in the same order as the mouse cornea. Quantitative results of the current mouse model study including the enhancement of gatifloxacin delivery may need to be calibrated for human case due to the differences between them.

In conclusion, we demonstrated that ultrasound treatment increased the trans-corneal delivery of gatifloxacin in the mouse cornea *in vivo*. Ultrasound waves of 1 MHz frequency, 1.3 W/cm^2^ intensity were applied onto the mouse cornea for 5 minutes, and then gatifloxacin ophthalmic solution was topically instilled. Ultrasound treatment increased gatifloxacin penetration into the mouse cornea and maintained gatifloxacin inside the cornea for an extended time duration. Therefore, ultrasound treatment might be beneficial for the therapeutic effects of gatifloxacin in the cornea.

## Methods

### Ultrasound treatment of the mouse eye

Ultrasound treatment on the mouse cornea was conducted by using a Sonitron GTS and a plane wave transducer (Nepagene Co., Ltd., Chiba, Japan). This transducer had a flat and round surface with 12 mm in diameter, and it operated at the frequency of 1 MHz in continuous mode. Ultrasound intensity could be adjustable from 0.1 to 5 W/cm^2^. Ultrasound treatment was conducted on the mouse cornea in both *ex vivo* and *in vivo* conditions. In case of *ex vivo* condition, an extracted fresh mouse eye ball was held in an acrylic holder filled with PBS, and oriented to point upward. The transducer tip was positioned from the top approximately 5 mm away from the cornea, and the treatment was conducted for 5 minutes. In case of *in vivo* condition, a mouse eye was held by using a mouse eye holder under gas anesthesia and temperature control. The eye was oriented to look upward, and the ultrasound transducer was positioned at approximately 5 mm away from the cornea. A viscous liquid (GenTeal Gel, Norvatis) was added in between the transducer tip and the eye as the coupling medium.

Various ultrasound intensities of 1.0 W/cm^2^, 1.3 W/cm^2^, 1.5 W/cm^2^ were applied onto mouse corneas for 5 minutes, to find the maximum ultrasound intensity applicable without damage. Right after the ultrasound treatment, 0.25% fluorescein solution (Fluorescite®, Alcon Korea Ltd.) was instilled on the corneas for 10 minute and then washed with PBS. Fluorescein solution usually does not penetrate the normal corneas because of tight junctions between corneal epithelial cells. However, if the tight junctions are lost or damaged, fluorescein solution penetrates and stains the damaged area^36^. After the fluorescein instillation, the ultrasound treated mouse corneas were examined by using a fluorescence macroscope (Motorized 16:1 Macroscope, Leica Z16 APO A). The light source was a mercury lamp with an excitation filter with 480 nm center wavelength and 40 nm bandwidth, and fluorescence images were acquired by using a camera with the combination of a dichroic mirror (505 nm long pass) and an emission filter (527 nm center wavelength/ 30 nm bandwidth). Corneal damage could occur by temperature rise during ultrasound treatment^23^ as well. Thus, temperature was measured in the region in contact with the viscous liquid (GenTeal Gel, Norvatis), which was used as the aqueous coupling medium for the propagation of the acoustic field, and by using a handheld infrared laser thermometer (Fluke Corporation, USA) during ultrasound treatment (ambient temperature: 24 °C).

Balb/c male mice, 6 weeks old, were used in for both the *in vivo* and *ex vivo* experiments. These mice were bred at the animal facility of POSTECH Biotech Center under specific pathogen free (SPF) conditions. All the experiment procedures were approved by POSTECH’s Institutional Animal Care and Use Committee (IACUC, approval number POSTECH-2015-0030-C1), and were conducted in accordance with the approved guidelines. Eight eyeballs from four mice were used for the *ex vivo* experiments and five eyes from five mice were used for the *in vivo* experiments.

### Reagents

Gatifloxacin 0.3% ophthalmic solution (Gatiflo, Handock Inc., Eun-Seong, Korea), was used as the drug to be delivered into the cornea. Gatifloxacin has the molecular weight of 375.4 g/mol and lipophilicity of 0.11^37^. Single drops of gatifloxacin ophthalmic solution were instilled onto the mouse eyes, incubated for 10 minutes, and then washed with PBS. In case of ultrasound treatment, gatifloxacin was instilled onto the cornea right after the treatment. The other procedures such as incubation and washing were the same as before.

### Two-photon microscopy (TPM) imaging of the mouse cornea

TPM was used to image gatifloxacin distribution in the mouse cornea based on the intrinsic fluorescence of gatifloxacin. A Leica SP5 multiphoton microscope system (Leica Microsystems, Wetzlar, Germany) equipped with a Ti-Sapphire laser (Chameleon Ultra II; Coherent, Inc., Santa Clara, CA) was used. Excitation wavelength was set at 790 nm for both gatifloxacin fluorescence and auto-fluorescence (AF) imaging. A 25× objective lens (HCX IRAPO L 25 × 0.95NA water immersion, Leica) was used. Emission light could be collected in 4 different spectral channels. En-face images in the x-y plane consisting of 512 × 512 pixels were typically acquired, and the field of view (FOV) was 310 μm × 310 μm. 3D imaging was conducted by acquiring multiple x-y plane images with the stepwise translation of the objective lens in the z direction by 2 μm. Excitation power for imaging gatifloxacin distribution in mouse corneas was approximately 22.3 mW at the sample. Emission light was collected by using 4 spectral channels, and images were generated by separating and color-coding channel 1 (390 nm ~ 410 nm) for SHG from collagen in the stroma and the other 3 channels (455 nm ~ 680 nm) for AF or gatifloxacin fluorescence. Imaging speed was 0.2 frames/s. To monitor the temporal changes of 3D gatifloxacin distribution in the same mouse corneas, time-lapse TPM imaging was conducted every 20 minutes for 1 hour starting right after 10-minute incubation.

### Statistical Analysis of TPM images

3D TPM images of gatifloxacin instilled mouse corneas were analyzed to calculate the depth profiles of average fluorescence intensity. Because the surface of mouse cornea was curved, a relatively flat central region of the cornea was selected as the region of interest (ROI). The ROI was approximately 129 μm × 129 μm in the x-y plane. This ROI was sub-divided into 7 × 7 sub-regions and the average intensity was calculated in each sub-region. Intensity profiles of the cornea was calculated by averaging those of individual sub-regions.

Because TPM images of the gatifloxacin instilled mouse cornea had both gatifloxacin fluorescence and AF components, the AF component needed to be subtracted to get the depth profile of gatifloxacin fluorescence intensity only. The AF component was measured by TPM imaging of the mouse cornea without gatifloxacin instillation. An average intensity profile of the AF component was obtained by repeating the measurement for 3 mouse corneas and by combining their depth profiles. The depth profile of gatifloxacin fluorescence intensity only was obtained by subtracting the average AF profile from the intensity profile of the gatifloxacin instilled mouse cornea.

## Supplementary information

En-face TPM video of a gatifloxacin instilled ex vivo mouse cornea after 10-minute gatifloxacin incubation

En-face TPM video of an ultrasound treated and gatifloxacin instilled ex vivo mouse cornea after 10-minute gatifloxacin incubation

En-face TPM video of a gatifloxacin instilled in vivo mouse cornea after 10-minute gatifloxacin incubation

En-face TPM video of an ultrasound treated and gatifloxacin instilled in vivo mouse cornea after 10-minute gatifloxacin incubation

Supplementary information for ultrasound-assisted gatifloxacin delivery in mouse cornea, in vivo

## Data Availability

The data generated during the current study are available from the corresponding author on reasonable request.
