# Supplementary material for: Ultrasound-assisted gatifloxacin delivery in mouse cornea, in vivo
Source: Sci Rep. 2019 Oct 29;9:15532. doi: 10.1038/s41598-019-52069-w (PMC6820539; doi:10.1038/s41598-019-52069-w)
Supplement: Supplementary file 5 — Supplementary information for ultrasound-assisted gatifloxacin delivery in mouse cornea, in vivo [file 41598_2019_52069_MOESM5_ESM.pdf]

Supplementary Information

Ultrasound-assisted gatifloxacin delivery in mouse cornea, *in vivo*

Uk Jegal[1], Jun Ho Lee[1], Jungbin Lee[1], Hyerin Jeong[3], Myoung Joon Kim[3,] & Ki Hean Kim[1,2,*]

Affiliations:

[1]Department of Mechanical Engineering, Pohang University of Science and Technology, 77 Cheongam-ro, Nam-gu, Pohang, yeoungbuk 37673, Rep. of Korea

[2]Division of Integrative Biosciences and Biotechnology, Pohang University of Science and Technology, 77 Cheongam-ro, Nam-gu, Pohang, yeoungbuk 37673, Rep. of Korea

[3]Department of Ophthalmology, Ulsan College of Medicine, Asan Medical Center, 88 Olympic-ro 43-gil, Songpa-gu, Seoul 05505, Rep. of Korea

*Corresponding author: Ki Hean Kim (kiheankim@postech.ac.kr)

Contents

List of Supplementary Videos

**Supplementary Video 1**	En-face TPM video of a gatifloxacin instilled *ex vivo* mouse cornea after 10-minute gatifloxacin incubation
**Supplementary Video 2**	En-face TPM video of an ultrasound treated and gatifloxacin instilled *ex vivo* mouse cornea after 10-minute gatifloxacin incubation
**Supplementary Video 3**	En-face TPM video of a gatifloxacin instilled *in vivo* mouse cornea after 10-minute gatifloxacin incubation
**Supplementary Video 4**	En-face TPM video of an ultrasound treated and gatifloxacin instilled *in vivo* mouse cornea after 10-minute gatifloxacin incubation

Supplementary Video & Legends

Supplementary Video 1: En-face TPM video of a gatifloxacin instilled *ex vivo* mouse cornea after 10-minute gatifloxacin incubation. (Corresponding to **Fig. 2(a-c)**)
The image size consists of 512 × 512 pixels covering a field-of-view (FOV) of 310 μm × 310 μm with a stepwise increment of 2 μm along the z-axis. Imaging speed was 0.77 frames/s. Color coding for emission fluorescence was as follows: 455 ~ 680 nm (*green*).

Supplementary Video 2: En-face TPM video of an ultrasound treated and gatifloxacin instilled *ex vivo* mouse cornea after 10-minute gatifloxacin incubation. (Corresponding to **Fig. 2(d-f)**)

The image size consists of 512 × 512 pixels covering a field-of-view (FOV) of 310 μm × 310 μm with a stepwise increment of 2 μm along the z-axis. Imaging speed was 0.77 frames/s. Color coding for emission fluorescence was as follows: 455 ~ 680 nm (*green*).

Supplementary Video 3: En-face TPM video of a gatifloxacin instilled *in vivo* mouse cornea after 10-minute gatifloxacin incubation. (Corresponding to **Fig. 3(a-c)**)
The image size consists of 512 × 512 pixels covering a field-of-view (FOV) of 310 μm × 310 μm with a stepwise increment of 2 μm along the z-axis. Imaging speed was 0.77 frames/s. Color coding for emission fluorescence was as follows: 455 ~ 680 nm (*green*).

Supplementary Video 4: En-face TPM video of an ultrasound treated and gatifloxacin instilled *in vivo* mouse cornea after 10-minute gatifloxacin incubation. (Corresponding to **Fig. 3(d-f)**)
The image size consists of 512 × 512 pixels covering a field-of-view (FOV) of 310 μm × 310 μm with a stepwise increment of 2 μm along the z-axis. Imaging speed was 0.77 frames/s. Color coding for emission fluorescence was as follows: 455 ~ 680 nm (*green*).
